# In Silico Docking, Resistance Modulation and Biofilm Gene Expression in Multidrug-Resistant *Acinetobacter baumannii* via Cinnamic and Gallic Acids

**DOI:** 10.3390/antibiotics11070870

**Published:** 2022-06-28

**Authors:** Neveen A. Abdelaziz, Walid F. Elkhatib, Mahmoud M. Sherif, Mohammed A. S. Abourehab, Sara T. Al-Rashood, Wagdy M. Eldehna, Nada M. Mostafa, Nooran S. Elleboudy

**Affiliations:** 1 Department of Microbiology and Immunology, Faculty of Pharmacy, Ahram Canadian University, Sixth of October City 12451, Egypt; neveen.abdelaziz@acu.edu.eg (N.A.A.); mahmoud.sherif@acu.edu.eg (M.M.S.); 2 Department of Microbiology and Immunology, Faculty of Pharmacy, Ain Shams University, African Union Organization St., Abbassia, Cairo 11566, Egypt; nooran.elleboudy@pharma.asu.edu.eg; 3 Department of Microbiology and Immunology, Faculty of Pharmacy, Galala University, New Galala 43727, Egypt; 4 Department of Pharmaceutics, Faculty of Pharmacy, Umm Al-Qura University, Makkah 21955, Saudi Arabia; maabourehab@uqu.edu.sa; 5 Department of Pharmaceutical Chemistry, College of Pharmacy, King Saud University, Riyadh 11451, Saudi Arabia; salrashood@ksu.edu.sa; 6 Department of Chemistry, School of Biotechnology, Badr University in Cairo, Cairo 11829, Egypt; wagdy2000@gmail.com; 7 Department of Pharmaceutical Chemistry, Faculty of Pharmacy, Kafrelsheikh University, Kafrelsheikh 33516, Egypt; 8 Department of Pharmacognosy, Faculty of Pharmacy, Ain Shams University, Cairo 11566, Egypt; nadamostafa@pharma.asu.edu.eg

**Keywords:** *Acinetobacter baumannii*, resistance modulation, cinnamic acid, gallic acid, biofilm

## Abstract

Despite the mounting global burden of antimicrobial resistance (AMR), the generation of new classes of effective antimicrobials still lags far behind. The interplay between multidrug resistance and biofilm formation in *Acinetobacter baumannii* has drastically narrowed the available therapeutic choices. The use of natural compounds holds promise as an alternate option for restoring the activity of existing antibiotics and attenuating virulence traits through reduced biofilm formation. This study aimed to evaluate the modulatory effect of combining cinnamic and gallic acids at ½MIC with various antibiotics against multidrug-resistant (MDR) *A. baumannii* clinical isolates as well as study the effect on the expression of the biofilm-associated genes (*bap*, *csuE*, *ompA*) via quantitative, real-time PCR. Combining cinnamic or gallic acid with imipenem, amikacin or doxycycline resulted in significant reduction of resistance (*p* < 0.05). On the contrary, no effect was recorded when both acids were combined with levofloxacin, and only cinnamic acid had a synergistic effect with colistin. The transcriptomic changes of biofilm-related genes in the presence of gallic acid at ½MIC were compared with untreated control samples. The fold expression values proved that gallic acid substantially down-regulated the respective genes in all five strong biofilm formers. Molecular docking studies of gallic and cinnamic acids on target genes revealed good binding affinities and verified the proposed mechanism of action. To the best of our knowledge, this is the first report on the effect of gallic acid on the expression of *bap*, *csuE* and *ompA* genes in *A. baumannii*, which may permit its use as an adjunct anti-virulence therapeutic strategy.

## 1. Introduction

“The clinical pipeline of new antimicrobials is dry” reported the WHO in November 2021 [1]. Despite the desperate need for novel antimicrobials in response to the pressing threat of antimicrobial resistance, none of the 43 antimicrobials presently being developed can face resistant bacteria topped by multidrug-resistant (MDR) Gram-negative bacteria and carbapenem-resistant *Acinetobacter baumannii* (CRAB) [2,3]. Antimicrobial resistance is reflected in longer hospitalization periods, elevated health care financial burdens, more severe complications and higher mortality rates [4,5]. It also casts a shadow over medical advancements such as chemotherapy, organ transplantation and other surgeries due to the risk of sepsis with difficult management [6]. Moreover, the problem of antimicrobial resistance is aggravated in resource-limited countries as well as in high-risk groups, including neonates [7]. A little less than one third of neonates suffering from bacteremia secondary to septic pneumonia die in spite of receiving antibiotic treatment [7].

Nearly all the antibiotics introduced in the past decades are mere variants of those discovered in the 1980s [8,9]. Restoring the activity of currently used antibiotics against bacterial pathogens is one of the futuristic approaches developed in the face of antimicrobial resistance [10]. A plethora of research is now dedicated to complementing antibiotics with natural compounds to reverse resistance [11,12,13,14,15,16]. The combination of antibiotics with natural products may not only circumvent resistance, but also decrease the dose used, consequently, reducing side effects [17,18]. Plant-derived compounds are ideal candidates due to their efficacy and considerably low side effects [19,20,21,22]. In phenolics, multiple mechanisms of antibacterial activity have been described; some compounds act by destabilizing cell membranes, thus, helping the internalization of antibiotics [23,24,25]. Others act by inhibiting efflux pumps or disrupting biofilms [26,27]. Biofilms are some of main players in the development of resistance in all MDR pathogens, with *A. baumannii*, one of the most notorious, nosocomial pathogens, being no exception [28,29]. Intriguingly, *A. baumannii* forms biofilms at a rate approaching 90%, which is the highest among pathogens [30,31]. Numerous virulence factors contribute to *A. baumannii* biofilm formation, mainly biofilm-associated protein (*bap*), the outer membrane protein A (*ompA*) and chaperon-usher pilus (*csu*) [32]. *Bap* is a sizable cell surface protein essential for intercellular communication and biofilm formation [33]. *OmpA* is humbler in size yet is *A. baumannii*’s main porin functioning in adherence, invasion, cytotoxicity and biofilm formation [34]. Pakharukova et al. reported that *csuA* deletion mutants are incapable of forming biofilms on abiotic surfaces, signifying that *csuA* is essential for the initial steps of biofilm formation [35]. Research on the antibiofilm properties of plant phenolics disclosed promising activities which affect the bacterial regulatory mechanisms, leading to biofilm suppression without any effect on bacterial growth [36]. Gallic and cinnamic acids are aromatic polyphenols present in a variety of fruits, vegetables and herbs. They have become more alluring to biologists by virtue of their myriad biological activities, and, on top of this, their antimicrobial and immunomodulatory effects [37,38]. However, most studies investigated their antibacterial activities against standard strains, food-borne pathogens and food-spoiling bacteria [39]. In light of this, the present study aims to investigate the resistance modulatory effect of cinnamic and gallic acids combined with various antibiotics on MDR *A. baumannii* clinical isolates as well as study the effect of gallic acid on the transcription of biofilm-related genes (*bap*, *csuE*, *ompA*) and its verification with in silico studies.

## 2. Results

### 2.1. Antimicrobial Synergistic Activity of Cinnamic and Gallic Acids

**Figure 1:**
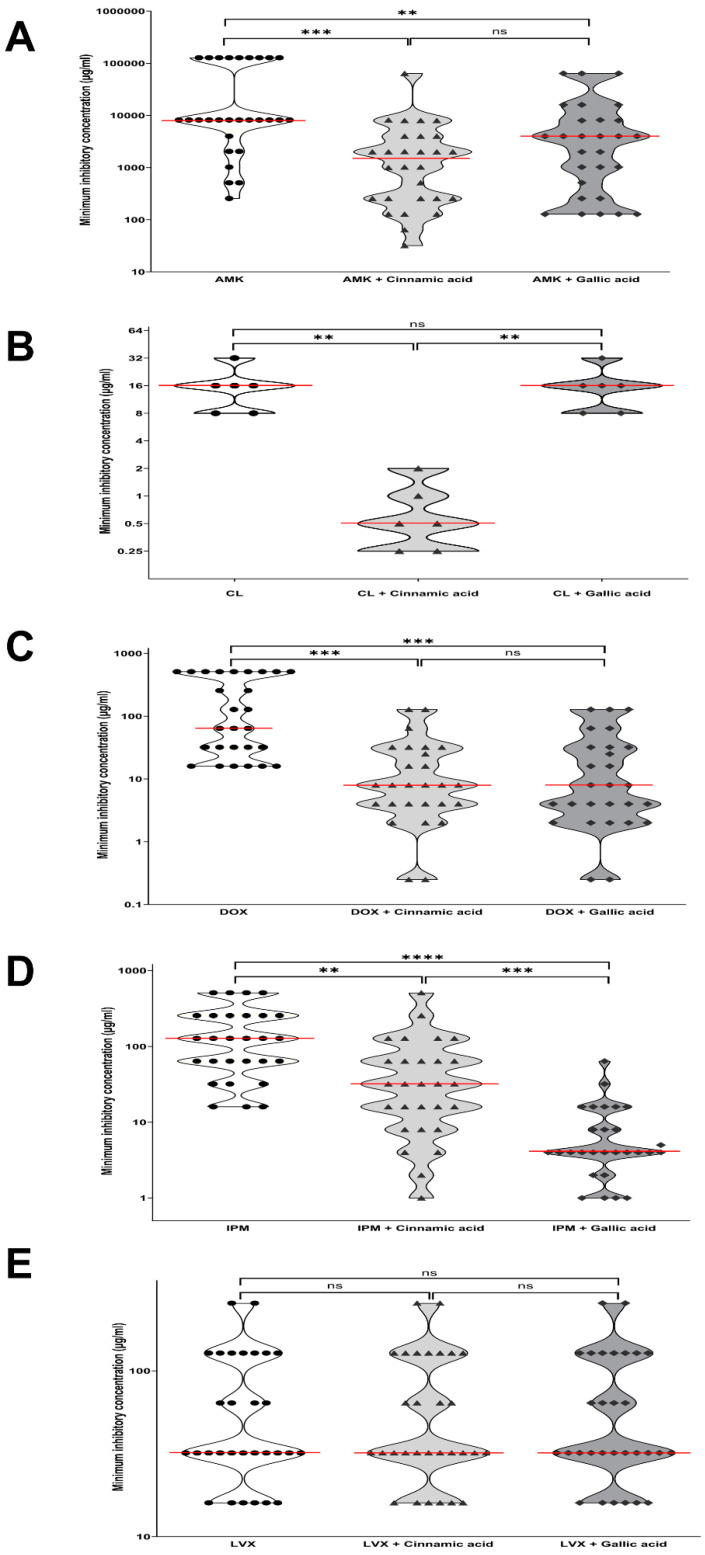
Violin plots showing MICs of the selected 30 MDR isolates against (**A**) amikacin, (**B**) colisitin, (**C**) doxycycline, (**D**) imipenem and (**E**) levofloxacin in presence/absence of cinnamic or gallic acids. *p*-values, **: *p* < 0.01, ***: *p* < 0.001, ****: *p* < 0.0001, ns: not significant (*p* > 0.05).

Gallic and cinnamic acids at ½MIC showed variable modulatory effects on resistance to the tested antibiotics. Combining cinnamic acid with colistin, imipenem, amikacin or doxycycline resulted in a significant reduction of resistance (*p*-value = 0.0059, 0.0088, <0.0001 and <0.0001, respectively; Figure 1). On the other hand, although all 30 tested MDR *A. baumannii* isolates were resistant to levofloxacin, no significant modulation of resistance was reported with either gallic or cinnamic acids. It is worth noting that the combination of colistin with cinnamic acid reversed the resistance of five test isolates to sensitive (MICs ranging from 0.25 to 1 µg/mL) and one to intermediate (MIC = 2 µg/mL; Supplementary Table S1). Likewise, the doxycycline resistance in 12 out of the 27 isolates was reversed to sensitive (MICs ranging from 0.25 to 4 µg/mL) and in 6 to intermediate (MIC = 8 µg/mL). Two imipenem-resistant isolates became sensitive (MICs = 1–2 µg/mL), and two became intermediate (MIC= 4 µg/mL) after adding cinnamic acid, while only one amikacin-resistant isolate became intermediate when combined with cinnamic acid (MIC = 32 µg/mL).

Vis-à-vis the ½MIC of gallic acid, it reverted 14 of the 27 doxycycline-resistant isolates to sensitive (MICs ranging from 0.25 to 4 µg/mL) and three of them to intermediate (MICs = 8 µg/mL). Likewise, reversion occurred in 7 of the 28 imipenem-resistant isolates, causing them to become sensitive (MICs ranging from 0.5 to 2 µg/mL), and 11 became intermediate (MICs = 4 µg/mL). Nevertheless, no significant effect was observed on any of the colistin-resistant and levofloxacin-resistant isolates (Figure 1).

Comparing the synergistic effects of the two phenolic acids showed that, though neither of them modulated resistance to levofloxacin, gallic acid had a superlative effect on imipenem resistance compared to cinnamic acid, with a statistically significant difference (*p* = 0.0007), while cinnamic acid had a superlative effect on colistin resistance with a statistically significant difference (*p* = 0.0059). In contrast, a non-statistically significant difference was detected between the modulatory effects of cinnamic and gallic acids on doxycycline (*p* ≥ 0.9999) and amikacin (*p* = 0.4002).

### 2.2. Effect of Gallic Acid (½MIC) on Expression of Biofilm-Related Genes

RT-qPCR was used to evaluate the transcriptomic changes of biofilm-related genes (*bap*, *csuE*, *ompA*) in the presence of gallic acid at ½MIC compared with untreated control samples.

**Figure 2:**
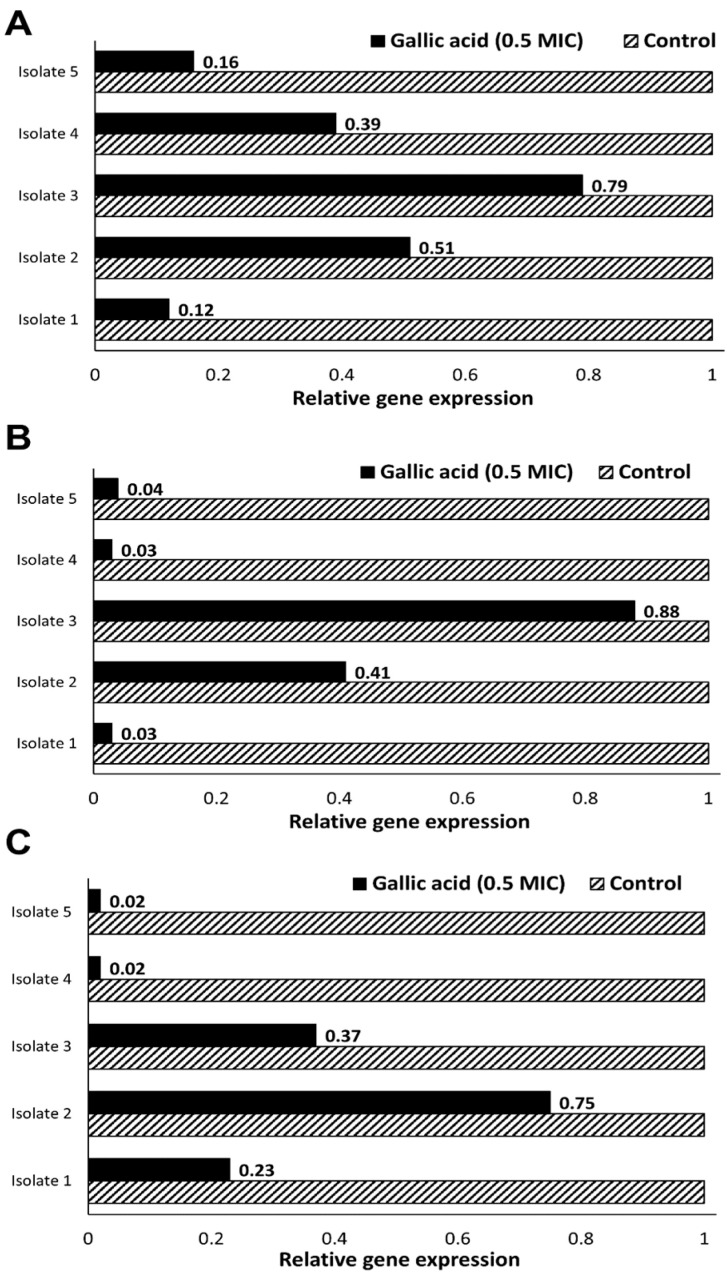
Relative expression of (**A**) *bap*, (**B**) *csuE* and (**C**) *ompA* genes in presence of gallic acid at ½MIC compared with untreated control samples.

The fold expression values proved that gallic acid substantially down-regulated biofilm-forming genes (*bap, csuE*, *ompA*) in all five strong biofilm formers. As shown in Figure 2A, the expression of the *bap* gene was significantly down-regulated by the effect of the ½MIC of gallic acid (*p* = 0.0078). While the fold expression fell to very low levels in isolates 1 and 5 (0.12 and 0.16, respectively), it dropped by only 20% in isolate 3. The expression of the *csuE* gene was also significantly affected by treatment with gallic acid (*p* = 0.0125; Figure 2B). The gene was almost unexpressed in isolates 1, 4 and 5 and was expressed at less than half its value in untreated samples by isolate 2; however, minimal effect was observed in isolate 3. Figure 2C shows that the treatment nearly inhibited the expression of the *ompA* gene in isolates 4 and 5 and had a variable inhibitory effect in the other three isolates (*p* = 0.006).

Looking at isolate 1, treatment with gallic acid nearly silenced the expression of *bap* and *csuE* genes and lowered that of the *ompA* gene by approximately 80%. The expression of *bap* and *csuE* genes by isolate 2 fell to half its value in treated samples as compared to untreated ones; however, the expression of the *ompA* gene decreased to only 75%. Nevertheless, expression of *bap* and *csuE* genes by isolate 3 was least affected by gallic acid treatment, while the expression of *ompA* fell to almost one third. The highest inhibition of *csuE* and *ompA* genes was observed in isolates 4 and 5, which also showed a reduction in expression of the *bap* gene to 0.39- and 0.16-fold, respectively.

### 2.3. Effect of Gallic Acid (½MIC) on Growth Rate

**Figure 3:**
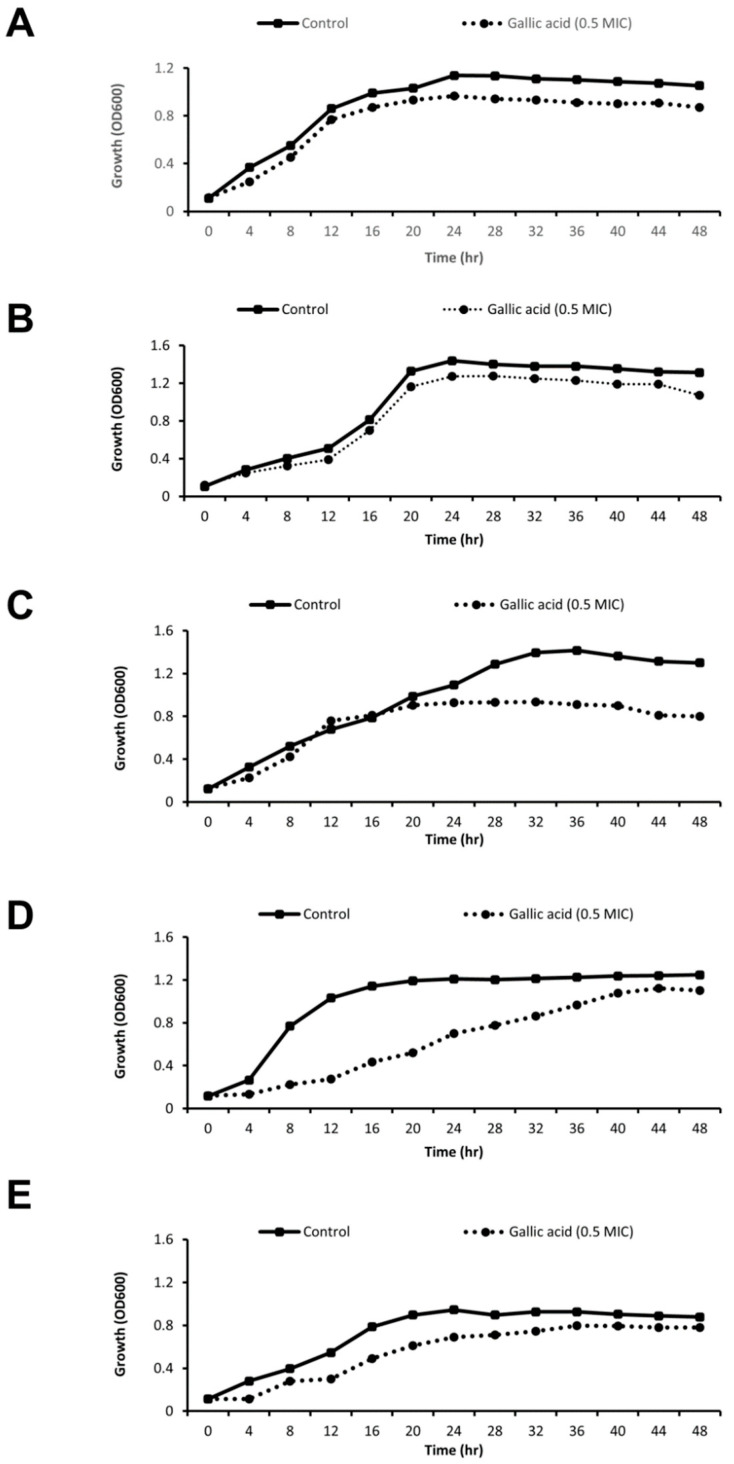
Bacterial growth curve of the five selected biofilm-forming *A. baumannii* isolates (**A**–**E**) in the presence of ½MIC of gallic acid along with the untreated growth controls.

Only slight growth pattern differences were observed between the control and some of the treated isolates, showing that sub-MIC gallic acid generally does not affect the viability of the tested strains during biofilm formation (Figure 3). This shows that the difference in gene expression is not due to the effect of gallic acid on isolates’ growth rate.

### 2.4. In Silico Molecular Docking Study on the Target Proteins

**Figure 4:**
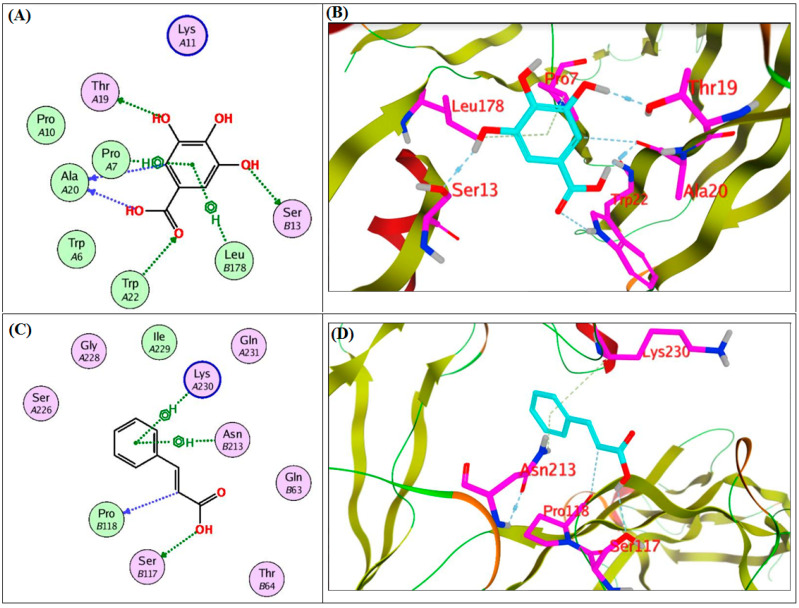
Binding diagrams of gallic (**A**,**B**) and cinnamic (**C**,**D**) acid into the active sites of CsuE protein.

**Figure 5:**
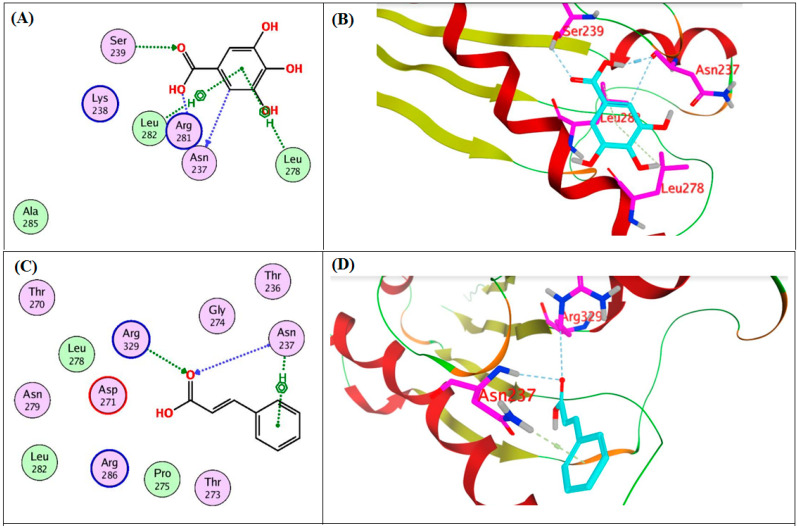
Binding diagrams of gallic (**A**,**B**) and cinnamic (**C**,**D**) acid into the active sites of OmpA protein.

The promising synergistic role of gallic and cinnamic acids in inhibiting the biofilm formation of *A. baumannii* encouraged us to conduct a docking study. The study aimed to identify the potential binding modes by which gallic and cinnamic exert their action. Therefore, the two acids were docked into the 3D coordinates of CsuE and OmpA proteins using the following PDB IDs: 6fjy and 3td3, respectively. The active site of the CsuE protein was determined using the MOE site finder, while the active site of OmpA was constructed as 4.5 Å surrounding the bound, co-crystalized glycine in the active site. The docking of the two acids (gallic and cinnamic) with the two proteins resulted in good, acceptable scores and strong binding modes. Interestingly, gallic and cinnamic achieved docking scores of −12.8 and −9.9 Kcal/mole with CsuE, while they achieved docking scores of −9.7 and −8.1 Kcal/mole with OmpA, respectively. As shown in Figure 4, gallic acid was found to interact with CsuE through hydrogen-bond interactions with Ser13, Thr19, Ala20 and Trp22, while it engaged in hydrophobic interactions with Pro7 and Leu178; similarly, cinnamic acid interacted with Ser117 and Pro118 through hydrogen bonds and with Asn213 and Lys230 through hydrophobic interactions. As depicted by Figure 5, the two compounds strongly interacted with the OmpA protein, in which gallic acid formed three hydrogen bonds with Asn237, Ser239 and Arg281 and two hydrophobic interactions with Leu278 and Leu282, while cinnamic acid formed two hydrogen bond interactions with Arg329 and Asn237 in addition to one hydrophobic interaction with Asn237.

## 3. Discussion

Antimicrobial resistance is the menace of twenty-first-century medical care. MDR *A. baumannii* displays extensive resistance to nearly all antibiotic classes, which made the WHO place it at the top of its agenda for research [40]. Accordingly, in this study we investigated the combinatory effect of the natural phenolic acids gallic and cinnamic acid and five antibiotics with distinct modes of action: two protein synthesis inhibitors (doxycycline and amikacin), an inhibitor of cell wall synthesis (imipenem), an inhibitor of cell proliferation through inhibition of DNA synthesis (levofloxacin) and colistin, which causes outer cell membrane disruption [41].

An intriguing finding of our binary combination study was that although combining cinnamic acid with colistin resulted in the restoration of the sensitivity of almost all resistant isolates, adding gallic acid to colistin-resistant isolates did not affect resistance. This may be attributed to the difference in mechanism of action. Colistin interacts with membrane lipopolysaccharides through replacing the Ca^2+^ and Mg^2+^ ions responsible for stabilizing the membrane. This results in loss of membrane integrity and cytoplasmic leakage followed by cell death [42]. A similar mechanism was proposed for gallic acid [43,44]. Functioning through similar mechanisms might be the reason for the lack of synergic effect [45]. Another explanation may be related to the antioxidant activity of gallic acid. Reactive oxygen species (ROS) are an important mechanism of killing by colistin; hence, co-administration of an antioxidant that quenches ROS increases persistent cells, as described by [46]. Collectively, the lack of change in the MICs of colistin with gallic acid may be attributed to the inverse mechanisms of action of gallic acid. Gallic acid may enhance permeability of colistin; however, its antioxidant activity may decrease the killing effect of colistin.

On the other hand, cinnamic acid, having three hydroxyl groups fewer, has been proposed to induce its membrane-damaging effect through altering the membrane lipid profile of Gram-negative bacteria, resulting in membrane acidification and protein denaturation [47].

Cinnamic and gallic acids have significantly modulated resistance to amikacin, imipenem and doxycycline. The acids’ effect on bacterial outer membranes might aid the penetration of the antibiotic molecules, elevating their intracellular concentrations in the face of resistance mechanisms [12,48]. Their inhibitory effect on efflux pumps might also be part of it [49,50]. Another proposed mechanism for the synergistic effect of phenolic acids on *A. baumannii* depends on their prooxidant potential. Being redox cyclers, phenolic acids increase production of reactive oxygen species assisting in cell death [51]. Several studies previously evaluated the synergism and modulatory effect of cinnamic and gallic acids with beta lactams and imipenem [52,53,54,55,56]. To the best of our knowledge, this is the first study that evaluates the modulatory effect of cinnamic acid with doxycycline; however, previous studies showed modulatory and synergistic effect between gallic acid and tetracycline against *Staphylococcus (S.) aureus* and *Escherichia (E.) coli* [57]. Additionally, gallic acid exhibited inhibitory effect on tetR and tetM efflux pumps that mediate tetracycline resistance in *Streptococcus* sp. [49]. Gallic acid, alkyl gallates and chitosan-based formulations of gallic acid can potentiate the antimicrobial activity of other antibiotics, including erythromycin, gentamicin, norfloxacin, ciprofloxacin, ampicillin, penicillin and oxacillin, via synergism [58]. The synergistic effect of cinnamic acid with amikacin against *Mycobacterium tuberculosis* and *Mycobacterium avium* was described by [59]. Similarly, [60] described the synergistic effect between cinnamic acid and amikacin against *E. coli* and *S. aureus;* however, there was no effect against *Pseudomonas (P.) aeruginosa*. On the other hand, gallic acid enhanced gentamycin activity against *S. aureus* and showed synergistic effect with amikacin against *E. coli*, as described by [61] and [62], respectively. It is noteworthy that sub-MICs of gallic acid showed a superlative modulatory effect with imipenem compared to cinnamic acid. We hypothesize that the divalent cation chelation activity of gallic acid may affect the activity of metallo-β-lactamases (MBLs) by zinc chelation, leading to the MBLs’ inactivation [63,64].

Although all the test *A. baumannii* isolates were resistant to levofloxacin, resistance was not affected by gallic or cinnamic acid at the tested concentrations. In the same vein, Lima et al. investigated the effect of gallic acid, caffeic acid and pyrogallol on the antibacterial activity of norfloxacin against Gram-negative (*E. coli* and *P. aeruginosa*) and Gram-positive (*S. aureus*) clinical isolates [61]. They reported that gallic acid enhanced antibacterial effect only against *S. aureus* [61].

Biofilm formation is one of the pivotal virulence factors and resistance enhancers in *A. baumannii* [65,66]. Hence, it has become imperative to develop entities with antibiofilm activities [67]. In our previously published work [68], we investigated the antibiofilm activities of cinnamic and gallic acids at ¼MIC and ½MIC concentrations, and results showed that gallic acid had a superlative antibiofilm effect against strong, biofilm-forming *A. baumannii* isolates. Consequently, in this study we investigated the effect of gallic acid at ½MIC on the expression of biofilm-related genes (*bap*, *csuE*, *ompA*). In order to rule out the effect of gallic acid on the growth rate of the isolates, a growth rate analysis in the absence and presence of ½MIC of gallic acid was conducted; results showed that gallic acid at this sub-MIC concentration generally did not affect the viability of the tested strains during biofilm formation.

Our results showed that gallic acid at ½MIC significantly down-regulated the expression of three of the key genes involved in biofilm formation by *A. baumannii* which are *bap*, *csuE* and *ompA*. This can be postulated as one of the factors contributing to its antibiofilm activity. Different natural products down-regulated expression of critical genes for biofilm formation in *Listeria monocytogenes* and *Pseudomonas aeruginosa*, as described by [69]. Additionally, melittin significantly down-regulated *bap* gene expression in *A. baumannii* [70]. Likewise, Kang et al. observed that the expression of the *mdoH* gene by *Shigella flexneri* was inhibited by the effect of gallic acid and concluded that gallic acid inhibited biofilm formation in *Shigella flexneri* through influencing the expression of the gene [71].

Computational studies of natural products have become indispensable for identifying possible mechanisms of action [72,73,74]. Based upon the performed in silico study, gallic and cinnamic acids showed the ability to strongly interact with the two selected proteins, CsuE and OmpA, achieving acceptable docking scores and a strong interaction pattern. These acceptable scores were achieved through the establishment of many hydrophobic and hydrogen-bond interactions. Thus, the observed strong binding interactions validated their activities and suggested possible mechanisms of action.

To the best of our knowledge, this is the first report on the effect of gallic acid on expression of *bap*, *csuE* and *ompA* genes in *A. baumannii*.

## 4. Materials and Methods

### 4.1. Antibiotics, Plant-Derived Compounds and Media

Amikacin was purchased from Eipico Co., Tenth of Ramadan City, Egypt; imipenem from Merck & Co., Kenilworth, NJ, USA; colistin, doxycyclin and levofloxacin from Sedico Co., Giza, Egypt. Cinnamic and gallic acids were obtained from Loba Chemie, Boisar, India, and dissolved in dimethyl sulfoxide DMSO (Fisher Scientific, Fair Lawn, NJ, USA) and distilled water, respectively. Cation-adjusted Mueller Hinton broth (CAMHB) and trypticase soy broth (TSB) were from Hi-Media, Mumbai, India.

### 4.2. Acinetobacter baumannii Clinical Isolates

In this study, we used thirty clinical MDR *Acinetobacter baumannii* isolates fully characterized in our previous work [68]. Their resistance profile is described in Table 1.

### 4.3. Antibiotic-Resistance-Modulating Effect of Cinnamic and Gallic Acids

MICs of five test antibiotics with different mechanisms of action, amikacin, imipenem, colistin, doxycycline and levofloxacin, were evaluated in the absence and presence of a sub-inhibitory concentration of cinnamic or gallic acids (½MIC determined in our previous work [68]) via broth microdilution technique [75]. Briefly, serial dilutions of the test antibiotics were prepared in cation-adjusted Mueller Hinton broth, cinnamic acid or gallic acid was added at its sub-inhibitory concentration (½MIC), then the plates were incubated. The MICs of the antibiotics were determined from rows containing only antibiotics. The modulatory effect was expressed in terms of the modulation factor. Modulation factors were evaluated as specified by [76] where a modulation factor value of 2 or higher indicates a biologically significant modulatory effect.

Modulation factor = MIC of antibiotic/MIC of antibiotic in presence of gallic or cinnamic acid(1)

### 4.4. Quantitative, Real-Time PCR

The effect of gallic acid at ½MIC on the expression of biofilm-associated genes (*bap*, *csuE*, *ompA*) was evaluated in five *A. baumannii* strong biofilm producers from our previous study [68]. All 5 isolates were resistant to imipenem, amikacin, doxycycline and levofloxacin, and only 2 exhibited reduced susceptibility to colistin. RT-qPCR was conducted as follow: First, the isolates were inoculated into TSB with or without gallic acid (½MIC) in 96-well, polystyrene, flat-bottom microtiter plates. The plates were incubated at 37 °C for 24 h. Cells were recovered by centrifugation at 3000 rpm for 5 min. Total RNA of biofilms in cell pellets was extracted by using Absolutely RNA Miniprep kit (Agilent, Santa Clara, CA, USA). Next, total RNA was reverse transcribed into cDNA by using TOPscript™ cDNA synthesis kit (Enzynomics, Republic of Korea). Gene expression was quantified via real-time PCR by using TOPreal™ qPCR 2X PreMIX SYBR Green with low ROX (Enzynomics, Republic of Korea) and the primers which were previously reported by [77]. In both gallic-acid-treated and untreated samples, 16S rRNA was used as a housekeeping gene [69]. Primer sequences are demonstrated in Table 2.

Relative fold gene expression method was used to analyze the expression of the biofilm genes s according to the melting curve [69]. Cycle threshold (CT) values were estimated by real-time PCR Applied Biosystems StepOne™ instrument (Foster City, CA, USA), then relative fold gene expression was calculated as follows:

Δ CT (Sample or Control) = CT (sample or control) − CT (housekeeping gene)(2)

ΔΔ CT = Δ Ct Sample − Δ CT control (3)

Relative fold gene expression = 2^−ΔΔCt^ (4)

The relative fold gene expression is the fold change compared to the untreated isolates which are assigned a value of 1. A change in gene expression is considered significant when there is a minimum of two-fold change [78].

### 4.5. Effect of Gallic Acid (½MIC) on Growth Rate

To confirm that gallic acid at ½MIC has no inhibitory effect on isolates’ growth, the 5 selected biofilm formers were subjected to a growth rate analysis in the presence of gallic acid at ½MIC [79]. In brief, 20 µL of an 18 h culture of each isolate was adjusted to 0.5 McFarland standard, then diluted to 200 µL with tryptic soy broth (TSB) in 96-well plates. Incubation was performed at 37 °C for 24 h. Growth was observed turbidimetrically by measuring the OD600 using ELx800, Biotek (Winooski, VT, USA) every 4 h for 48 h. Gallic acid was added at ½MIC, and measurements of growth inhibitory activity were performed as triplicates using untreated growth controls.

### 4.6. In Silico Molecular Docking Study

The docking study was conducted to demonstrate the binding affinities of the tested compounds to the active sites of the protein [80,81]. The study was performed using Molecular Operating Environment (MOE 2019.02) software [82,83]. The X-ray crystal structures of CsuE and OmpA proteins were downloaded from the protein data bank using the PDB IDs 6fjy and 3td3, respectively. At the beginning, the hydrogens and charges of the receptors were optimized using AMBER10: EHT embedded in MOE software. The active site of CsuE protein was determined using MOE site finder, while the active site of OmpA was constructed as 4.5 Å surrounding the bound, co-crystalized glycine in the active site. Gallic and cinnamic acids were sketched using the 2D builder of MOE 2019 and converted to 3D structures using the same software. After that, they were docked in the binding site of CsuE and OmpA proteins using triangular matcher and London dg as a placement and scoring methods, respectively. At last, 2D and 3D interaction diagrams were generated by MOE to analyze the docking results.

### 4.7. Statistical Analysis

All analyses were carried out using R statistical platform (https://www.r-project.org, accessed on 30 April 2022) in R-studio, version 1.4.1106. In quantitative variables, normality assumption was tested using chi-squared goodness-of-fit test. For normally distributed data, *t*-test and ANOVA were used to compare the means of two groups and multiple groups, respectively. Kruskal–Wallis (KW) test was used to compare the medians for non-normally distributed data. Mann–Whitney and Tukey’s HSD tests were applied as post hoc tests using Bonferroni correction method for multiple comparisons in the Kruskal–Wallis and ANOVA tests, respectively. For all statistical analyses, *p*-values < 0.05 were considered statistically significant.

### 4.8. Ethical Approval

The protocol of this study was approved to be compliant with the regulations of the ethical committee of the Faculty of Pharmacy, Ahram Canadian University. The collected isolates were obtained as such from the microbial isolate depository of El Demerdash Hospital, Cairo, Egypt, without any interaction with patients; thus, informed consents were inessential.

## 5. Conclusions

In this study we described the complementary effect of cinnamic and gallic acids combined with various antibiotics on MDR *A. baumannii* clinical isolates. A statistically significant reduction in resistance was attained by the combination of cinnamic or gallic acid with imipenem, amikacin or doxycycline. Conversely, no effect was recorded when both acids were combined with levofloxacin, and only cinnamic acid had a synergistic effect with colistin. Moreover, our results showed that gallic acid at ½MIC significantly down-regulated the expression of three of the key genes involved in biofilm formation by *A. baumannii*, which are *bap*, *csuE* and *ompA*. This was further verified by the in silico molecular docking study, in which gallic and cinnamic acids achieved acceptable docking scores and a strong interaction pattern with the two selected proteins CsuE and OmpA.

## Figures and Tables

**Table 1:** Resistance profiles of the 30 MDR clinical *Acinetobacter baumannii* isolates.

Antibiotic	Number of Resistant Isolates (%)
Levofloxacin	30 (100)
Imipenem	28 (93.3)
Amikacin	28 (93.3)
Doxycycline	27 (90)
Colistin	6 (20)

**Table 2:** Primer sequences for the genes evaluated.

Gene	Primer	
*bap*	Forward Reverse	TGCTGACAGTGACGTAGAACCACA TGCAACTAGTGGAATAGCAGCCCA
*csuE*	Forward Reverse	CATCTTCTATTTCGGTCCC CGGTCTGAGCATTGGTAA
*ompA*	Forward Reverse	GTTAAAGGCGACGTAGACG CCAGTGTTATCTGTGTGACC
16S rRNA	Forward Reverse	ACCGTCAAGGGACAAGCA GGGAGGCAGCAGTAGGGA

## Data Availability

All data are in the manuscript and its supplementary file.

## Supplementary Materials

The following supporting information can be downloaded at: https://www.mdpi.com/article/10.3390/antibiotics11070870/s1, Table S1: MICs of the selected 30 MDR isolates against colistin (CL), imipenem (IPM), doxycycline (DOX), amikacin (AMK) and levofloxacin (LVX) in presence/absence of cinnamic or gallic acids.
