# Supplementary material for: In Silico Docking, Resistance Modulation and Biofilm Gene Expression in Multidrug-Resistant Acinetobacter baumannii via Cinnamic and Gallic Acids
Source: Antibiotics (Basel). 2022 Jun 28;11(7):870. doi: 10.3390/antibiotics11070870 (PMC9311515; doi:10.3390/antibiotics11070870)
Supplement: Supplementary file 1 [file antibiotics-11-00870-s001.zip › antibiotics-1786221-supplementary.pdf]

Supplementary Table S1. MICs of the selected 30 MDR isolates against colisitin (CL), imipenem (IPM), doxycycline (DOX), amikacin (AMK), and levofloxacin (LVX) in presence/ absence of cinnamic or gallic acids.

	MIC (µg/ml)														
**Isolate**	CL	CL+ Cinnamic acid	CL+ Gallic acid	IPM	IPM+ Cinnamic acid	IPM+ Gallic acid	DOX	DOX+ Cinnamic acid	DOX+ Gallic acid	AMK	AMK+ Cinnamic acid	AMK+ Gallic acid	LVX	LVX+ Cinnamic acid	LVX+ Gallic acid
3	0.5	0.5	0.5	512	128	4	256	4	16	512	256	128	128	128	128
16	16	0.5	16	0.5	0.5	0.5	64	4	4	2048	128	256	32	32	32
30	0.5	0.5	0.5	256	128	4	64	4	4	8192	256	128	128	128	128
48	8	0.25	8	512	32	4	512	128	32	8192	2024	512	16	16	16
2	0.5	0.5	0.5	512	256	4	128	4	4	512	512	128	128	128	128
35	0.5	0.5	0.5	256	128	4	512	4	4	8192	1024	128	32	32	32
40	0.5	0.5	0.5	128	64	4	16	4	4	8192	128	4000	64	64	64
13	0.5	0.5	0.5	256	64	16	128	8	8	8192	2000	128	128	128	128
18	0.5	0.5	0.5	128	32	32	32	2	2	8192	1000	8192	64	64	64
4	16	0.25	16	256	128	8	16	2	2	8192	8000	4000	256	256	256
1	8	2	8	64	16	4	512	32	64	8192	256	4000	128	128	128
11	0.5	0.5	0.5	64	4	2	512	32	128	8192	128	2000	32	32	32
3	32	1	32	64	32	1	32	2	2	8192	2000	2000	32	32	32
76	0.5	0.5	0.5	64	1	2	32	0.25	2	8192	64	4000	32	32	32
97	0.5	0.5	0.5	128	8	8	512	32	32	8192	4000	8000	32	32	32
79	0.5	0.5	0.5	32	32	1	512	8	8	128000	8192	1024	64	64	64
30	0.5	0.5	0.5	512	512	64	512	32	64	4000	32	4000	128	128	128
87	0.5	0.5	0.5	16	16	0.5	256	8	32	128000	2000	16000	32	32	32
94	0.5	0.5	0.5	64	64	1	16	8	0.25	128000	8000	8000	16	16	16
35	16	0.5	16	16	8	8	512	128	128	2048	256	1024	16	16	16
20	0.5	0.5	0.5	32	32	4	64	16	16	256	256	256	32	32	32
5	0.5	0.5	0.5	256	64	16	16	8	4	128000	8000	64000	128	128	128
Sam 109	0.5	0.5	0.5	64	8	4	32	16	8	128000	2000	16000	16	16	16
G9	0.5	0.5	0.5	256	64	16	16	0.25	2	128000	4000	16000	256	256	256
50	0.5	0.5	0.5	16	4	4	0.5	0.5	0.5	1024	1024	1024	64	64	64
77	0.5	0.5	0.5	128	2	16	512	64	128	128000	64000	4000	16	16	16
88	0.5	0.5	0.5	128	16	4	32	8	0.25	128000	2000	64000	16	16	16
95	0.5	0.5	0.5	128	16	16	0.5	0.5	0.5	128000	4000	64000	32	32	32
